# Performance of ivisen IA‐1400, a new point‐of‐care device with an internal centrifuge system, for the measurement of cardiac troponin I levels

**DOI:** 10.1002/jcla.23747

**Published:** 2021-03-17

**Authors:** Ha Nui Kim, Soo‐Young Yoon

**Affiliations:** ^1^ Department of Laboratory Medicine Korea University Guro Hospital Seoul Korea

**Keywords:** cardiac troponin, cardiac troponin I, i‐SENS, ivisen IA‐1400, point‐of‐care

## Abstract

**Background:**

We present the analytical performance of the ivisen IA‐1400, a new point‐of‐care device that features a characteristic built‐in centrifuge system, to measure blood cardiac troponin I (cTnI) levels.

**Methods:**

Whole blood and plasma samples obtained from patients who visited Korea University Guro Hospital were used to analyze measurement range, cross‐reactivity, interference, and sensitivity and specificity. We performed a correlation analysis of the ivisen IA‐1400 versus the Access AccuTnI+3 immunoassay using the UniCel™ DxI 800 platform and the PATHFAST™ hs‐cTnI assay.

**Results:**

Within‐run precisions were classified as low, 9.8%; middle, 10.2%; and high, 8.5%. The limit of blank was 3.1 ng/L for plasma samples and 4.3 ng/L for whole blood samples. The limit of detection was 8.4 ng/L for plasma samples and 10.0 ng/L for whole blood samples, respectively. The limit of quantitation at a coefficient of variation of 20% and 10% was 19.5 ng/L and 45.5 ng/L for plasma samples, respectively. The comparative evaluation between the two other assays and ivisen IA‐1400 showed excellent correlation, with Spearman's correlation coefficients (R) of 0.992 and 0.985. The sensitivity and specificity of ivisen IA‐1400 using the optimum cut‐off value of 235 ug/L were 94.6% and 98.2%, respectively.

**Conclusion:**

The ivisen IA‐1400 showed acceptable and promising performance in cTnI measurements using whole blood and plasma samples, with limited information in the clinical performance. The flexibility for sample selection using the internal centrifugation system is the main advantage of this point‐of‐care device.

## Graphical abstract

- This study aims to introduce a new point‐of‐care device for measuring the blood cardiac troponin, ivisen IA‐1400, with a characteristic built‐in centrifuge system developed in South Korea.
- We obtained acceptable performance data of ivisen IA‐1400, including excellent correlation with Access AccuTnI+3 immunoassay using the UniCel^TM^ DxI 800 platform and PATHFAST^TM^ hs‐cTnI assay.

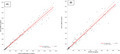

## 1 INTRODUCTION

The rapid and accurate diagnosis of cardiovascular diseases is essential for initiating appropriate and timely medical treatment, especially for life‐threatening emergencies, such as acute myocardial infarction (AMI). The World Health Organization incorporated the serial testing of cardiac biomarkers in the diagnostic criteria for AMI in 1986, along with a history of chest pain and changes on electrocardiograms^1^

A cardiac biomarker is a biochemical compound used to detect cardiac diseases such as AMI and myocardial injury.^2^ These compounds should be sensitive and specific to cardiac tissue, provide results with a short turnaround time (TAT), and be cost‐effective.^3^ After the first report of the use of a biochemical marker for myocardial injury in 1954,^4^ numerous new diagnostic marker proteins that aid the assessment of cardiac diseases and prediction of cardiovascular risk have been identified. The European Society of Cardiology/American College of Cardiology recommends the use of cardiac biomarkers for the diagnosis of myocardial injury, preferably cardiac troponin (cTn [I or T]). When the membranes of cardiac muscle cells are damaged, cTns are released into the circulation. Both cTnI and cTnT can be measured using commercially available analytical platforms.^5^ From the development of early monoclonal antibody‐based diagnostic immunoassays to recent high‐sensitivity cTnI and cTnT (hs‐cTnI and hs‐cTnT) assays, the limit of detection (LoD) has been lowered, although the values are highly variable among various hs‐cTnI assays, ranging from 0.009 ng/L to 2.5 ng/L.^6^

To provide earlier treatment for AMI, there remains a need for a more rapid and efficient measurement of cTns. One strategy is the development and use of point‐of‐care (POC) testing platforms.^7^ The cTns measurements obtained using central laboratory equipment are more sensitive than POC measurements.^5^ However, POC devices can deliver rapid results, within 30 minutes near the bedside, whereas cTn assays performed in the central laboratory usually take longer because of sample transport, handling, and pretreatment. Fast diagnosis from rapid TAT results can reduce the length of hospital stay and overall hospital costs.^8^, ^9^ These devices employ more user‐friendly systems that can be operated by less‐skilled personnel. Therefore, POC devices can be beneficial in situations where there are no skilled medical technologists or 24‐hour laboratory operations are impossible. These advantages of POC assays have led to the development of cTn assays with high analytical quality and a shorter TAT.

Herein, we present the analytical performance of a newly developed POC device, the ivisen IA‐1400 (i‐SENS, Seoul, South Korea), in terms of imprecision, linearity, cross‐reactivity with interferences, sensitivity, and specificity, with a correlation analysis for comparison to preexisting devices. As the ivisen IA‐1400 can process both whole blood and plasma samples and we aimed to evaluate the correlation between the two sample types as well.

## 2 MATERIALS AND METHODS

### 2.1 ivisen IA‐1400

**FIGURE 1:**
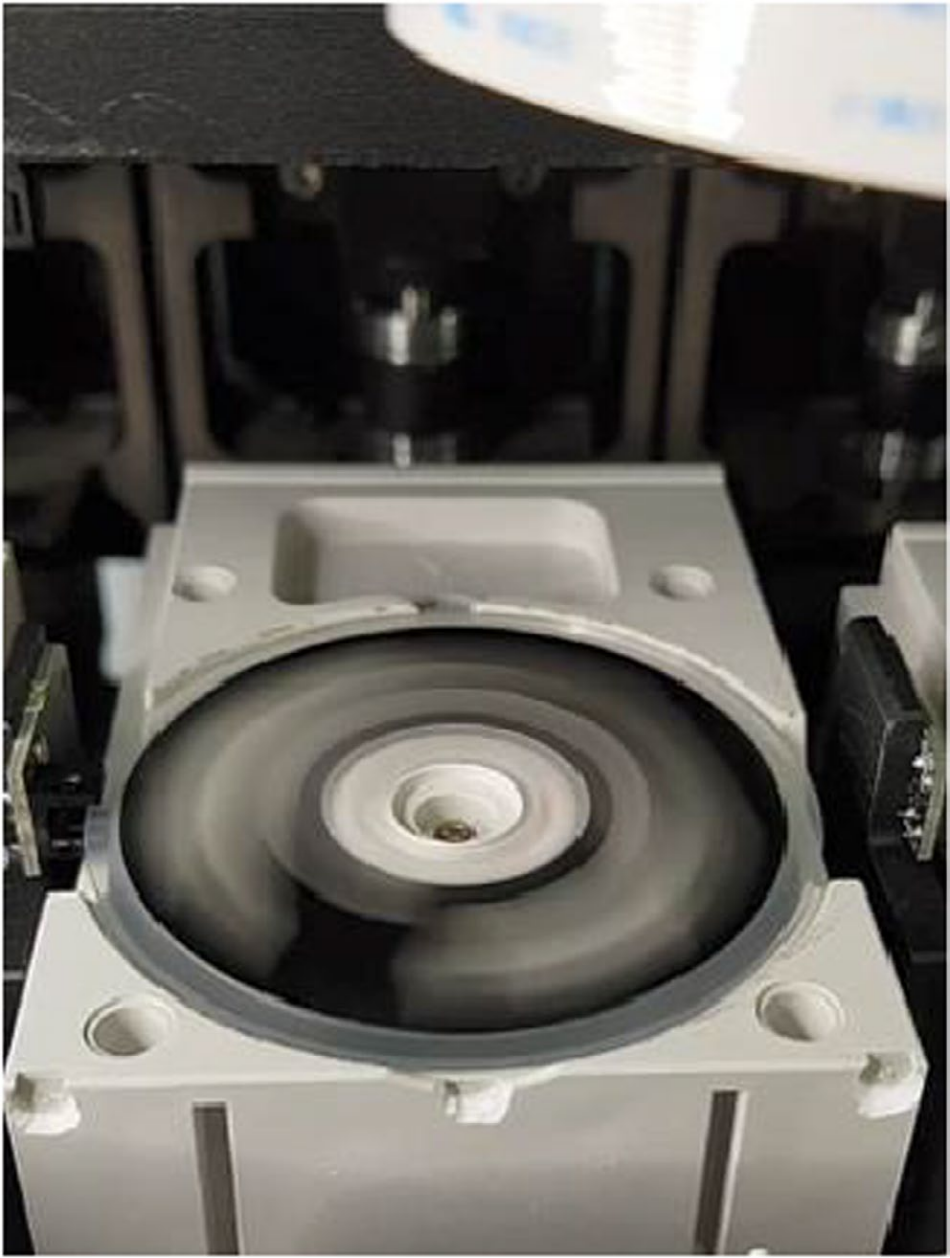
Internal centrifuge in ivisen‐IA 1400 system that rotates the cartridge at 4000 rpm in 2 min. This image is provided from i‐SEN, Inc. with permission

The ivisen IA‐1400 system is a compact, fully automated, bench‐top immunoassay analyzer with a modular configuration that offers random access ability to perform one to four tests simultaneously. The system rotates the cartridge at 4,000 rpm, allowing the direct measurement of whole blood samples without sample pretreatment required for plasma separation, and consequently providing rapid quantitative measurements of cardiac biomarkers in less than 17 min. To utilize whole blood samples in preexisting POC immunoassay devices, cTnI values can be obtained after software correction by using each sample's externally measured hematocrit value.^10^, ^11^ In contrast, the ivisen IA‐1400 system itself has a built‐in internal centrifuge as shown in Figure 1 and does not need to measure hematocrit separately. Therefore, the measurement process is simple and has a shorter TAT than other POC devices, even when whole blood samples are used.

### 2.2 Samples and study protocols

We enrolled 872 leftover ethylenediaminetetraacetic acid (EDTA)‐whole blood samples from patients who were requested to undergo both complete blood count and cTnT tests in Korea Guro University Hospital from January 2019 to December 2019. For the cTnT measurement, the hs‐cTnT assay (Elecsys Troponin T hs STAT, Roche Diagnostics, Mannheim, Germany) was performed on the cobas 8000 e602 analyzer (Roche Diagnostics) in our laboratory. Whole blood samples were stored at −80°C, and thawed before evaluation. Before the evaluation, samples with evidence of severe hemolysis, coagulation, contamination, or insufficient quantity were excluded.

Plasma samples obtained from the EDTA‐whole blood samples were used to verify the analytical measurement range (AMR), interference with cross‐reactivity testing, and performance evaluation for the sensitivity, specificity, and correlation analysis. Whole blood and plasma samples were used to determine the limit of blank (LoB), LoD, and limit of quantitation (LoQ), with a comparative correlation analysis performed by sample type (whole blood vs. plasma). All analytical procedures were conducted in accordance with the Clinical and Laboratory Standards Institute (CLSI) guidelines. This study was approved by the institutional review board of Korea University Guro Hospital (IRB no. 2019GR0018).

### 2.3 Imprecision study

The imprecision study of the ivisen IA‐1400 was performed in accordance with the CLSI guidelines EP05‐A2.^12^ The quality control (QC) materials, Liquichek^TM^ Cardiac Markers Plus Control LT (Bio‐rad Laboratories, Headquarters, Hercules, CA, USA) were used to determine imprecision. Three different levels of QC materials were measured twice a day with a minimum interval of 2 hours between measurements, in duplicate, for 20 consecutive days. This process was performed on three different lots at each QC level to define the between‐lot precision. To achieve reproducibility, three different QC levels were tested in triplicate twice a day for 5 consecutive days in two separate laboratories. Results including mean, standard deviation (SD), and coefficients of variation (CVs) were calculated for each of the three different lots and QC levels.

### 2.4 Estimation of LoB, LoD, and LoQ for analytical sensitivity

The LoB, LoD, and LoQ were calculated based on the CLSI guidelines EP17‐A2.^13^ All measurements were made using two different cartridges, one for the plasma samples and another for the whole blood samples. The greater values of LoB, LoD, and LoQ are reported for the overall study as recommended in CLSI guidelines EP17‐A2. The LoB is the highest apparent analyte concentration when replicates of a blank sample containing no analyte are tested and defined by the relation LoB=meanblank +1.645(SD_blank_).^14^ Using cTnI‐free plasma and whole blood sample as a blank, the LoB was calculated from values obtained in 60 repeated measurements of two lots. The highest LoB value was used to obtain the LoD. To calculate the LoD and LoQ, samples showing high cTnI concentrations were pooled and serially diluted to reach their target concentrations. A total of seven pooled samples with different concentrations were prepared. LoD, the lowest analyte concentration determined by using both measured LoB and test replicates of a low concentration of analyte, is defined by the formula LoD = LoB + 1.645(SD _low concentration sample_). For the LoD estimation, the process was repeated for 5 days in five pooled samples and measured three times each at the estimated LoD concentration. The LoQ is the lowest concentration at which predefined goals for bias and imprecision are met. To calculate the LoQ, two pooled samples with estimated concentrations that resulted in CVs of 10% and 20% were measured three times for 5 days.

### 2.5 AMR and hook effect

The AMR or linearity of the method was determined according to the CLSI guidelines EP06‐A.^15^ Using Seracon^TM^ cTnI‐free human plasma as a negative‐reference material and human cardiac troponin I‐T‐C complex (Hytest Ltd., Turku, Finland) as a positive‐reference material, 10 pools were prepared by serial dilution. Six of the high‐concentration pools exceeding the upper measurable range (>10,000 ng/L) were used to examine the high‐dose hook effect. All high‐dose hook samples were measured in triplicate on the ivisen IA‐1400 cTnI cartridge.

### 2.6 Cross‐reactivity and interference

A total of 24 interfering substances (acetaminophen, acetylsalicylic acid, allopurinol, ampicillin, ascorbic acid, atenolol, caffeine, captopril, digoxin, dopamine, erythromycin, furosemide, methyldopa, niphedipine, phenytoin, theophylline, verapamil, bilirubin‐conjugated, bilirubin‐free, hemoglobin, human anti‐mouse antibodies [HAMA], rheumatoid factor, and triglycerides) and eight cross‐reacting materials (actin protein, creatine kinase myocardial band, human skeletal muscle troponin I, C, and T, myoglobin, myosin, and tropomyosin) were tested for interference and cross‐reactivity for four pools with negative, low, medium, and high cTnI concentrations according to the CLSI EP07‐02 guidelines.^16^ All pooled samples with various materials were measured in triplicate and the average value was obtained to calculate bias.

### 2.7 Method and sample type comparison

The results obtained from the ivisen IA‐1400 were compared with those of two preexisting hs‐cTnI assays using 111 samples: Access AccuTnI+3 (AccuTnI+3) immunoassay using UniCel^TM^ DxI 800 platform (Beckman Coulter Inc., Fullerton, CA, USA), and PATHFAST^TM^ hs‐cTnI assay (Mitsubishi Medience, Tokyo, Japan) using plasma samples in accordance with CLSI guidelines EP09‐A3.^17^ The epitope peptides of AccuTnI+3 used for the cTnI measurement were the same as those used in ivisen IA‐1400. A sample type comparison study of the whole blood and plasma samples was conducted of 39 paired samples using the mean value of the duplicates. A Passing‐Bablok regression analysis was performed to define the relationship and agreement between devices in the comparison analysis. A Bland‐Altman analysis was also performed of the comparative evaluation of the significant differences between the sample types.

### 2.8 Optimal cut‐off value for sensitivity and specificity

The diagnostic performance of the ivisen IA‐1400 for predicting AMI was briefly evaluated. Measurements of cTnI were performed using residual samples from 415 patients with suspected AMI who visited Korea University Guro Hospital. The recruited samples were further classified as “non‐AMI sample” (341 samples) or “AMI sample” (74 samples), based on the clinicians’ diagnosis according to the universal guidelines for diagnosing AMI.^18^ The optimal cut‐off value, which maximizes the sensitivity and specificity, was obtained from the best receiver‐operating characteristic curve (ROC) cut‐off value in the ROC analysis.

### 2.9 Statistical analysis

The statistical analysis was performed using Analyse‐it Software (Analyse‐it Software, Leeds, UK) and Microsoft Excel version 2016 (Microsoft Corporation, Redmond, WA, USA).

### 2.10 Patient and public involvement

No patients or members of the public were involved in the design of this study.

## 3 RESULTS

### 3.1 Imprecision

Mean, SD, and CV values were obtained using three different QC materials (level 1, low; level 2, middle; and level 3, high) and three lots (Table 1). Within‐run precisions combining the results of the three lots were as follows: low, 9.5%; middle, 10.2%; and high, 8.5%. Between‐lot precisions were as follows: low, 6.0%; middle, 4.6%; and high, 4.8%. A total reproducibility was 10.1%, 12.2%, and 9.9% at the low, middle, and high levels, respectively.

**TABLE 1:** Imprecision study of ivisen IA‐1400 using Liquichek^TM^ Cardiac Markers Plus Control LT (Bio‐rad) of level 1, 2, and 3

Control	Mean (ng/L)	Repeatability (%)	Between‐lot CV (%)	Reproducibility (%)
Level 1	17.6	9.5	6.0	10.1
Level 2	415.6	10.2	4.6	12.2
Level 3	2085.1	8.5	4.8	9.9

Abbreviation: CV, Coefficient of variation.

### 3.2 Analytical sensitivity: LoB, LoD, and LoQ

Both plasma and whole blood samples were used for the LoB, LoD, and LoQ analyses (Table 2). The LoB for lots 1 and 2 were 3.1 and 2.4 ng/L for plasma samples, and 4.3 and 2.8 ng/L for whole blood samples, respectively. The LoD for lots 1 and 2 were 8.4 and 7.1 ng/L for plasma samples, and 10.0 and 6.4 ng/L for whole blood samples, respectively. The LoQ values were calculated at CVs of 20% and 10%. The LoQ at CVs of 20% and 10% were 14.4 and 45.5 ng/L for plasma samples and 28.6 and 57.2 ng/L for whole blood samples, respectively, in lot 1. In lot 2, the LoQ at CVs of 20% and 10% were 19.5 and 39.1 ng/L for plasma samples and 15.6 and 31.1 ng/L for whole blood samples, respectively. The greater of two values were reported as the LoB, LoD, and LoQ for the ivisen IA‐1400. No statistically significant differences in the LoQ were observed between the plasma and whole blood samples (*P* > 0.05). The LoB, LoD, and LoQ profiles using plasma samples of the AccuTnI+3, PATHFAST, and ivisen IA‐1400 are summarized in Table 3.^19^, ^20^, ^21^ The data, including the 99^th^ percentile upper reference limit (URL) for the AccuTnI+3 and PATHFAST were provided by their respective manufacturers.

**TABLE 2:** Summary of LoB, LoD, and LoQ in measurement of cTnI using ivisen IA‐1400 by sample type and lot. Bold letters indicate the reported value for overall study

Sample type	Plasma		Whole blood	
Cartridge lot	Lot 1	Lot 2	Lot 1	Lot 2
LoB (ng/L)	**3.1**	2.4	**4.3**	2.8
LoD (ng/L)	**8.4**	7.1	**10.0**	6.4
LoQ at CV20% (ng/L)	14.4	**19.5**	**28.6**	15.6
LoQ at CV10% (ng/L)	**45.5**	39.1	**57.2**	31.1

Abbreviations: CV, coefficient of variationLoB, limit of blank; LoD, limit of detection; LoQ, limit of quantitation.

**TABLE 3:** Profiles of LoB, LoD, and LoQ at CVs of 20% and 10% of Access AccuTnI+3, PATHFAST^TM^ hs‐cTnI assay, and ivisen IA‐1400 using plasma samples

Method	LoB (ng/L)	LoD (ng/L)	LoQ at CV20% (ng/L)	LoQ at CV10% (ng/L)	99th percentile upper reference limit (ng/L)
Access AccuTnI+3	4.5	10.9	17.1	30.4	40
PATHFAST^TM^ hs‐cTnI	1.2	2.3	4.0	15.0	29.0
ivisen IA−1400	3.1	8.4	19.5	45.5	NA

Abbreviation: NA, not available.

### 3.3 Linearity and hook effect

According to the regression analysis of 10 pooled replicates, linearity was confirmed for the range of 10 ng/L (LoD value) to 10,000 ng/L. No hook effect was observed since there was no decrease in the measured value at high concentrations up to 36,700 ng/L.

### 3.4 Cross‐reactivity and interference

The calculated bias (%) at low and high concentrations of the eight cross‐reacting materials was less than 1%, suggesting no significant cross‐reactivity. None of the tested interfering substances showed bias greater than ±20%, except for the high level of rheumatoid factor (500 IU/mL). The level of the rheumatoid factor that met the bias of less than ±20% was 125 IU/mL at high concentrations and 250 IU/mL at low concentrations. Immunoassays using mouse antibodies are prone to interference from heterophilic antibodies. For the evaluation of HAMA, multiple types of HAMA materials from different manufacturers were used and the bias was less than ±20%. However, in patients with a history of exposure to mice or immunotherapy, the results should be interpreted cautiously.

### 3.5 Method and sample type comparison

**FIGURE 2:**
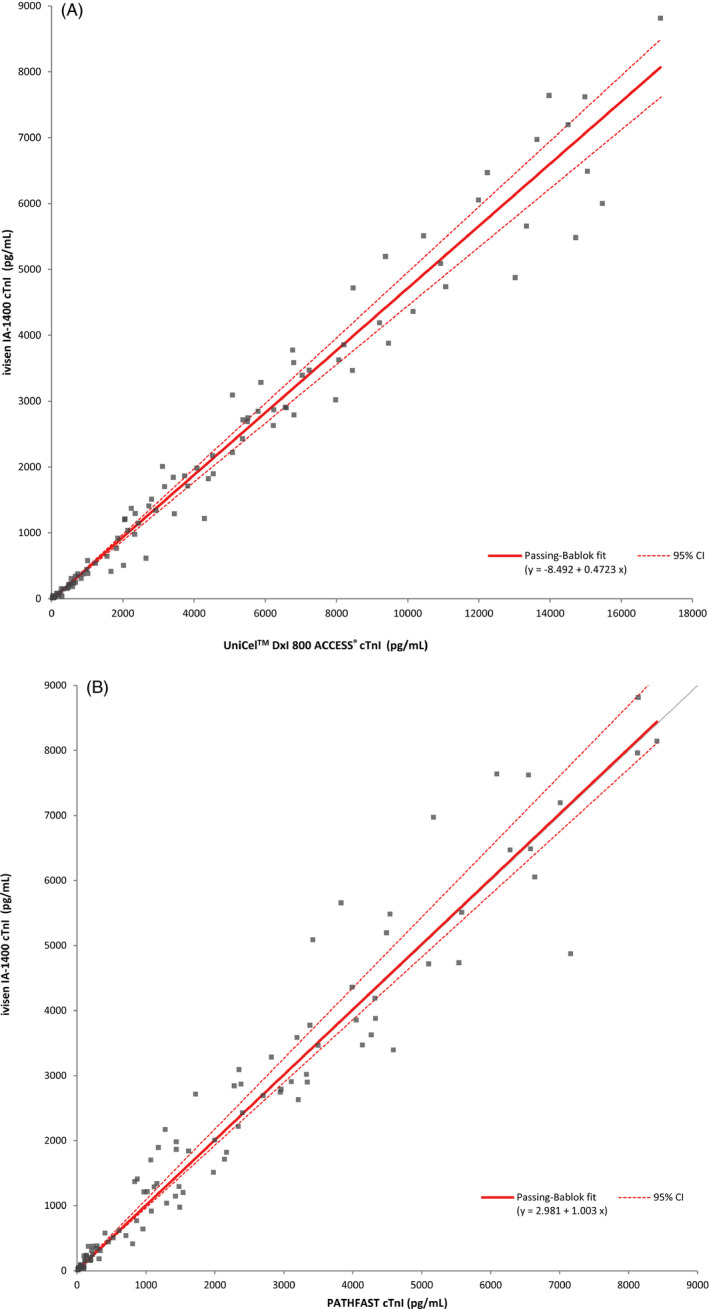
The comparative evaluation between Access AccuTnI+3 assay (A), PATHFAST^TM^ hs‐cTnI assay (B) and ivisen IA‐1400

**FIGURE 3:**
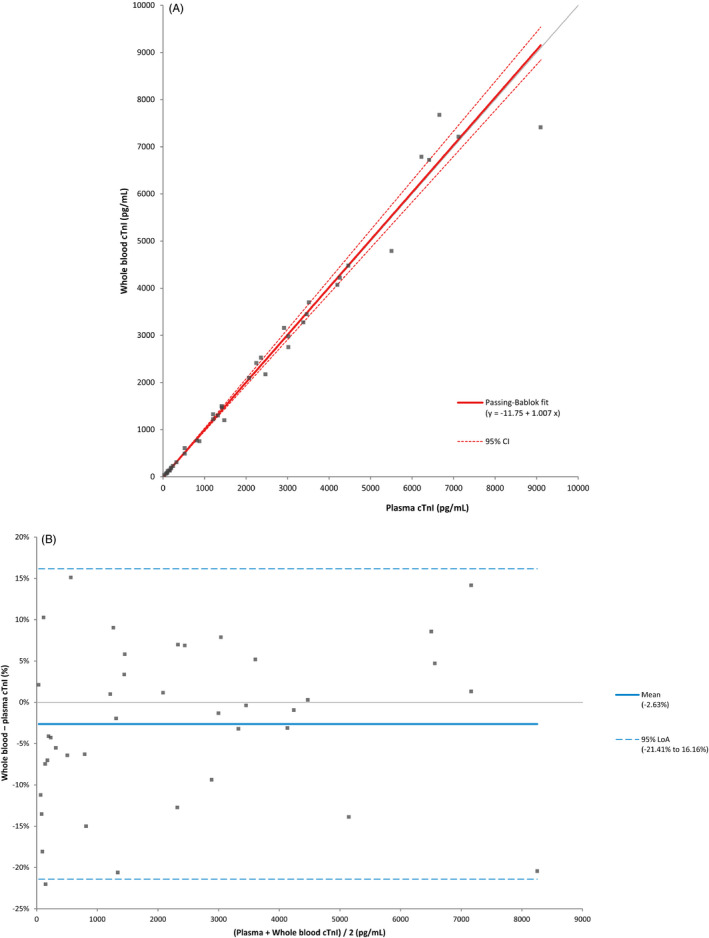
The results of correlation analysis between whole blood and plasma samples using ivisen IA‐1400 (A) Passing‐Bablock regression, (B) Bland‐Altman plot

The comparative evaluation of the AccuTnI+3, PATHFAST, and ivisen IA‐1400 using 111 plasma samples showed excellent correlation, with Spearman's correlation coefficient (R) values of 0.992 and 0.985, respectively (Figure 2, Table 4). The results of the correlation analysis between whole blood and plasma samples using the ivisen IA‐1400 are shown in Figure 3A. The correlation between the whole blood and plasma samples was excellent (R = 0.988), and no significant bias (−2.6%, *P* > 0.05) was observed in the Bland‐Altman plot (Figure 3B).

**TABLE 4:** Correlation coefficients (R) with 95% CI for cTnI measurement for methods and sample type

	R	Intercept (95% CI)	Slope (95% CI)
Access AccuTnI+3 vs. ivisen IA−1400	0.992	−8.492 (−32.37 to −1.697)	0.4723 (0.4512 to 0.4934)
PATHFAST^TM^ hs‐cTnI assay vs. ivisen IA−1400	0.985	2.981 (−1.765 to 19.89)	1.003 (0.9658 to 1.075)
Sample type (whole blood vs. plasma)	0.988	−11.75 (−27.45 to 1.370)	1.007 (0.9796 to 1.047)

Abbreviation: CI, confidence interval.

### 3.6 Optimal cut‐off values for sensitivity and specificity

The optimal cut‐off value derived from the ROC analysis of the ivisen IA‐1400 was established as 235 ng/L. In the two groups previously classified by diagnosis (74 AMI and 341 non‐AMI cases), the sensitivity and specificity of ivisen IA‐1400 at the value of 235 ng/L were 94.6% and 98.2%, respectively. The ROC curve showed an area under the curve of 0.998, confirming excellent discrimination.

## 4 DISCUSSION

POC platforms have the advantages in terms of the promptness of delivering information for patient care and decision‐making, especially in medical emergencies. However, the significances of these advantages need to be carefully evaluated due to the lower sensitivity of POC tests compared to that of central laboratories, especially in the early stages after symptom onset. According to a study conducted in an emergency department (ED), sensitivity was significantly lower in patients sampled <3 hours after symptom onset than in those sampled >3 hours after symptom onset.^5^ Another study indicated some discrepant results between the results of POC testing and those of laboratories, revealing six false negative results and three false positive results in a total of 189 samples in POC testing.^22^ As the cut‐off value was adjusted higher, the number of discrepancies decreased (only two false positives), while the correlation increased. The authors in this study described that POC testing should be considered to provide faster cTn results and that the continued use of POC testing could help increase ED throughput, and decrease wait times and lengths of stay. According to a research article that analyzed the effect of POC testing on actual cost, POC testing decreased the referral rate in patients without acute coronary syndrome and also achieved tangible reductions in costs.^23^ Therefore, to fully leverage of POC testing, an appropriate performance evaluation process is essential to ensuring acceptable quality of the information provided by POC testing.

This study evaluated the analytical performance of the ivisen IA‐1400 for taking cTnI measurements. The built‐in centrifuge is the most characteristic aspect of this new POC device, as it enables the convenient use of whole blood samples without a pretreatment process. However, the difference in sample type may affect cTnI levels. In the sample type correlation study, the results of the plasma and whole blood samples from the same patient demonstrated excellent correlation without significant difference, as shown in the Bland‐Altman plot. Therefore, the use of the ivisen IA‐1400 can reduce TAT by eliminating pretreatment time loss and increasing the flexibility in the choice of sample type.

Among the two types of hs‐cTn assays (hs‐cTnI and hs‐cTnT), hs‐cTnT assays are supplied by only one manufacturer (Roche Diagnostics) because of patent restrictions on the antibodies selected for the assay.^18^ The choice of whether to measure hs‐cTnI or hs‐cTnI appears to be a judgment based on the situation of each laboratory (e.g., depends on who the primary supplier to the local laboratory is) rather than clinical decisions.^21^ In our laboratory at Korea University Guro Hospital, the hs‐cTnT assay has been used since 2011. The ivisen IA‐1400 system showed acceptable sensitivity and specificity in the two populations (AMI vs. non‐AMI) diagnosed using the hs‐cTnT assay. In the precision study, acceptable repeatability, reproducibility, and between‐lot precision results (±10%) were achieved using three lots. The LoD values for the plasma and whole blood samples were below 10 ng/L, with no significant interference or cross‐reactivity. The results of the whole blood and plasma samples were significantly correlated, confirming the stable performance of the internal centrifuge, a special feature of the ivisen IA‐1400. The correlation study of the AccuTnI+3 and PATHFAST revealed a very strong correlation (R = 0.992 and 0.985, respectively). However, LoQ values at CVs of 20% and 10% were higher than those of the other two assays using plasma samples, especially when compared with the PATHFAST (at CV20%: ivisen IA‐1400, 19.5 ng/L, PATHFAST, 4.0 ng/L; and CV10%, 45.5 ng/L, 30.4 ng/L, respectively). The PATHFAST demonstrated a complete fulfillment of the analytical criteria for hs‐cTn assays, surpassing a CV of <10% at the 99^th^ URL and a CV of 5.1% at 29 ng/L.^24^ As the ivisen IA‐1400 was developed as a contemporary device, it is not appropriate to compare the values with the same standard as the PATHFAST hs‐cTnI assay.

There are some limitations to be mentioned in this study. For the qualified determination of the 99^th^ percentile URL for a cTn assay, an adequate reference population of at least 300 healthy individuals with an appropriate age, ethnicity, and sex is required.^25^ As this study used leftover whole blood samples from patients with various diseases other than AMI and other underlying medical conditions requiring cTn testing, the 99^th^ percentile URL could not be obtained. This value is crucial in evaluating the overall performance of cTn testing devices and determining whether the device can be classified as a contemporary or hs‐cTn assay. Although the sensitivity and specificity calculated from the optimum cut‐off value were acceptable, the information gained from the clinical performance data is of limited value. A follow‐up study with the study population of healthy individuals will be necessary to determine whether the ivisen IA‐1400 will achieve the ideal recommendation of a CV <10% at the 99^th^ percentile URL or a CV <20% as acceptable POC testing for clinical use. Second, although the comparison with the other two devices showed satisfactory results, there is a relatively insufficient number of samples in the lower range, which is important in clinical decision‐making process. As mentioned above, further studies need to be carried out using more data at the low cTnI concentrations to assure the analytical performance in the lower range. Another point to note is that mild discrepant CV values were observed between precision and LoQ analysis. Since the QC material was used in the precision analysis, not patient samples, it can be assumed that better CV values were obtained due to the difference in matrix.

In conclusion, a newly developed contemporary POC device for cTnI, the ivisen IA‐1400, showed acceptable and promising performance in cTnI measurements using whole blood and plasma samples, with respect to speed, imprecision, analytical sensitivity with specificity, and correlation. If the CV at the 99^th^ percentile URL is acceptable for clinical use as POC testing, the characteristic internal centrifuge system can be conveniently used in various situations where whole blood samples are used along with plasma samples.

## CONFLICT OF INTERESTS

None declared.

## Data Availability

The data that support the findings of this study are available from the corresponding author upon reasonable request.
